# LBA: Online Learning-Based Assignment of Patients to Medical Professionals

**DOI:** 10.3390/s21093021

**Published:** 2021-04-25

**Authors:** Hanan Rosemarin, Ariel Rosenfeld, Steven Lapp, Sarit Kraus

**Affiliations:** 1 Department of Computer Science, Bar-Ilan University, Ramat Gan 5290002, Israel; h.rosemarin@gmail.com (H.R.); zvikush9@gmail.com (S.L.); sarit@cs.biu.ac.il (S.K.); 2 Department of Information Science, Bar-Ilan University, Ramat Gan 5290002, Israel

**Keywords:** human-computer interaction, healthcare, smart environments

## Abstract

Central to any medical domain is the challenging patient to medical professional assignment task, aimed at getting the right patient to the right medical professional at the right time. This task is highly complex and involves partially conflicting objectives such as minimizing patient wait-time while providing maximal level of care. To tackle this challenge, medical institutions apply common scheduling heuristics to guide their decisions. These generic heuristics often do not align with the expectations of each specific medical institution. In this article, we propose a novel learning-based online optimization approach we term Learning-Based Assignment (LBA), which provides decision makers with a tailored, data-centered decision support algorithm that facilitates dynamic, institution-specific multi-variate decisions, without altering existing medical workflows. We adapt our generic approach to two medical settings: (1) the assignment of patients to caregivers in an emergency department; and (2) the assignment of medical scans to radiologists. In an extensive empirical evaluation, using real-world data and medical experts’ input from two distinctive medical domains, we show that our proposed approach provides a dynamic, robust and configurable data-driven solution which can significantly improve upon existing medical practices.

## 1. Introduction

The patients to medical professionals ratio has decreased over the years across the globe [1]. As a result, medical professionals are faced with an ever increasing dynamic flow of patients who present a wide variety of conditions, all of which seek high quality and fast medical attention. Due to the variability in patients’ conditions, as well as the limited availability of medical professionals and their own variability (i.e., domain of expertise and experience), an efficient patient to medical professionals assignment process is needed [2,3].

The patient to medical professional assignment problem is directed at getting the right patient to the right medical professional at the right time, given the medical institute’s objective and constraints. Specifically, given a patient’s arrival and the available medical staff, a decision has to be made as to when the patient should receive treatment and by which medical professional. With recent technological advances in healthcare and massive AI and sensor integration in the medical field we are seeing a growing transition to healthcare being provided by automated systems. For example, consider a patient entering an emergency department. Advanced sensors enable automated sensing of the patient’s health metrics and provide secure and privacy preserving access to her medical records. Given this automated assessment, the task of prioritizing the patient and assigning a suitable medical professional has to be properly addressed. In this work, we address this challenge with a novel learning-based approach. As of today, most patient to medical professional assignment processes are performed by people (e.g., by triage nurses in the emergency department) using conventional heuristics for online scheduling which may perform sub-optimally. These heuristics commonly focus on medically-established upper bounds (or deadlines) for the patient’s waiting time for treatment [4]. Specifically, they are aimed at minimizing these violations as exceeding these upper bounds may result in increased risk for adverse medical consequences.

Minimizing deadline violations need not necessarily best align with the medical institution’s (complex) objective. For example, in an Emergency Department (ED) setting, the ED is faced with multiple partially-conflicting objectives such as minimizing patients’ risk (e.g., allocating the most suitable physician) on the one hand and reducing wait time (e.g., assigning each patient to the first available physician) on the other. It is self-evident that considering only a subset of these objectives, as ED heuristics often do, will likely fall short of achieving the desired medical institution’s objective [5,6]. As such, decision makers can benefit from the use of an intelligent decision support system which is tailored for the entire set of objectives and constraints [7].

On top of the aforementioned complexities, additional two key requirement of the medical domain is flexibility and autonomy. Flexibility is needed to support different patient arrival flows and available medical staff (due to changing circumstances such as national holidays, global pandemic, etc.) and the varying nature of the institute’s objective (due to new regulations and managerial focuses). Thus, the desired approach has to exhibit both robustness to uncertainty and sufficient configurability to properly support different medical institutions over time. In addition, in the medical domain, decision makers have to maintain a certain degree of autonomy. For example, in most EDs, physicians may be asked to treat a certain patient at a given time. In other settings, a higher degree of autonomy may be required by the physicians such that each of them is provided with a list of possible patients. The physician is then allowed to prioritize the list at her own discretion. This list is often referred to as the medical professional’s “exposure list”. A representative example is the exposure of scans to radiologists for medical analysis. In both cases, a decision maker is allowed to ignore the recommendation of a decision support system, and, thus, the desired approach has to dynamically adapt and continue to provide high quality recommendations.

In this article, we propose a general-purpose learning-based approach we term Learning-Based Assignment (LBA), which can address both the aforementioned challenges and can be applied to the two medical assignment scenarios mentioned above. Our approach combines standard offline optimization techniques with machine learning to provide a dynamic, robust and configurable data-driven solution. Specifically, our approach starts with a specification of a medical assignment process, a set of metrics and a complex objective. These are translated into a formal optimization problem along with domain specific constraints. The optimization problem is then instantiated into small-scale scenarios, based on real-world data, which are solved using offline optimization techniques. The set of solutions is used to train a machine learning model, which is in turn used to supply a recommended assignment to the medical decision maker.

In an extensive empirical evaluation, using real-world data and medical experts’ input in two medical scenarios, we show that our proposed LBA approach can significantly improve the patient to medical professional assignment over established medical practices, which can translate into better health care for the greater good.

## 2. Related Work

To better understand the unique characteristics of patient–medical professional assignment problem, we next situate the problem within the existing literature and point to the central limitations of existing approaches. The assignment of patients to medical professionals can be seen as a special type of Job Shop Scheduling (JSS), which is a fundamental problem in computer science and operations research [8]. The scheduling of jobs to machines in JSS is analogous to the allocation of patients to medical professionals in a medical setting. JSS is also prevalent in multi-agent systems, such as multi-robot task-allocation [9,10], and in other batch allocation of tasks, such as in crowd-sourcing [11]. Similar to the medical settings we investigate in this article, JSS problems often face the challenge of properly addressing complex and partially conflicting objectives (i.e., translating a given objective into a suitable heuristic) as well as accounting for the varying capabilities of the machines (as in the case of unrelated parallel scheduling [8]). An additional challenge is the question of partial completion of tasks. For example, Zheng and Shroff [12] addressed the scheduling of computer jobs to a cloud cluster in a setting where tasks arrive online but give some partial value for partial execution. Naturally, in most medical settings, patients must be *fully* treated and therefore we do not allow for partial treatment. One exception to the above is the preemption case, where a medical professional is called to treat a higher priority patient in mid-treatment, which only occurs in extreme cases. The notion of preemption is not unique to the medical setting and was also investigated by Doucette et al. [13], when addressing the assignment of tasks to agents in an online fashion. Neither of the above studies addresses the possible differences in valuation and the completion rate of the agents/machines in contrast to how medical professionals vary (i.e., seniority and specialty). In addition, the medical domain introduces domain-specific challenges. As mentioned above, medical professionals require a certain degree of “freedom” in ignoring recommendations and/or prioritizing their tasks (unlike machines in the conventional JSS setting). To the best of our knowledge, this challenge has yet to be addressed in the literature.

From a machine learning perspective, task scheduling using classification was recently investigated (e.g., [14,15]). Most work in this realm focuses on solving *offline* problems with dependencies (e.g., temporal dependencies) and deadlines, while we focus on solving *online* problems of independent tasks (patients) where there is no strict deadline but rather a desired upper bound on the patient’s waiting time. More relevant to this work is the method in [16] which takes a Learning from Demonstration (LfD) approach—that is, learning human-quality heuristics based on demonstrations—to a scheduling problem without varying values for tasks. Similar to our proposed approach, the authors used a pairwise ranking function; however, while the authors tried to mimic a human-quality policy, we follow optimal solutions, thereby overcoming the inherent suboptimality of existing human-generated medical practices.

From the medical perspective, few works have addressed patient assignment concerns. Notably, Peretz et al. [17] focused on the nuclear medicine domain and took a two-stage stochastic integer programming approach to assign patients that require multi-step tests, e.g., a patient arrives with three tests to be performed that have to be performed sequentially, with the restriction that any individual task cannot be paused once it has begun. In the proposed model, once a patient’s tasks are assigned (possibly, in the future), they cannot be changed or interrupted, a constraint we do not have in most medical settings. There are also various techniques for allocating medical staff to shifts under different constraints [2]. Similarly, optimization methods have been developed and applied for emergency response systems (e.g., [18]). Several works have focused on minimizing the overall patient time in the ED. For example, Harzi et al. [19] used a mixed integer linear program approach to derive optimal scheduling of patients for a given scenario (specified by the patient’s arrival times and the number of physicians). Unfortunately, this approach is limited by the number of patients (the authors were unable to evaluate it with 25 patients or more). Moreover, this approach does not generalize to a dynamic online setting where patient arrivals are unknown in advance. Similarly, Luscombe and Kozan [20] modeled the ED environment as parallel machines flexible job shop scheduling problem which is solved using a tabu search approach. To the best of our knowledge, this line of research has not addressed, to date, the challenges outlined above in the online assignment of patients to medical professionals to date. Specifically, it is common in these works to assume that no preemptions are allowed and that all physicians are “identical” such that the specialty and experience of a physician is not considered when assigning a patient to a physician.

Our medical setting is centered around the assignment of patients to medical professionals. Assignment algorithms are generally divided into two categories: (1) *point-wise*, in which each possible option is given a score and the highest ranking option is selected; and (2) *comparison-based*, in which each pair (or list) of possible options are compared to each other providing a partial order among options. Prior work has shown that pairwise approaches work better in practice than point-wise approaches resulting in popular “Learning to Rank” algorithms such as RankNet, LambdaRank and LambdaMART [21] which are pairwise approaches. Drawing inspiration from the above algorithms, in this article, we propose the use of supervised machine-learning algorithms (e.g., deep neural networks) to train a pair-wise ranking function. Specifically, we develop a learning-based method to train a ranking function which is at the core of our approach. This method is inspired by the learning-to-ranking algorithm used in information retrieval [22]. In the information retrieval setting, the success of the trained heuristic is determined by point-wise evaluation. Namely, each prediction is scored independently from other predictions. However, in most medical settings, the score is determined by a series of sequential decisions and not by a point-wise scoring function.

Our approach also allows medical professionals to have a certain degree of autonomy as required. For example, the decision of which patients should appear in one’s exposure list may be viewed as choosing a list of recommendations to be presented. This problem is often addressed by recommender systems [23]. At the core of the classic recommendation setting, a recommender predicts the rating or preference that a user has over a list of items (e.g., what movies or TV shows will a user find interesting [24]). However, this classic setting has also been extended such that the recommender’s task is subject to a set of constraints and an optimization objective, similar to our setting. For example, Shani et al. [25] casted the recommendation problem as a Markov Decision Process (MDP) such that the long-term effects of each recommendation could be considered and the expected returns could be optimized. Similar optimization approaches have been proposed for movie recommendations [26,27], nutrition [28] and online dating [29,30], to name a few. To the best of our knowledge, none of the above works have addressed the domain specific challenges associated with medical assignments such as the time-sensitive nature of the process. From a technical perspective, common recommender systems do not seem to be applicable to our setting, as they consider each recommendation separately and locally. Simply put, they assume an interaction of a “single user” with a fixed set of items, whereas in our case a group of medical professionals jointly work on a shared dynamically changing set of tasks.

Decision support tools have been developed in the literature to improve the efficiency of healthcare delivery services such as improving scheduling of elective patients [31], nurse routing for home dialysis [32] and other home healthcare logistics [33], to name a few. Our work complements these efforts by addressing the unique aspects of online, near real-time, assignment of patients to medical professionals.

## 3. The LBA Approach

To tackle the patient to medical professional assignment challenge outlined above, we propose a novel machine learning-based approach we term as Learning-Based Assignment (LBA). LBA is aimed at approximating the idealized optimal offline assignment (i.e., an omniscient scheduler), which is informed of the patients’ arrival distribution, their characteristics and the available staff in advance. Our setting is *online* and *event-driven*, namely, when a new medical task arrives (i.e., a new patient arrives at the ED or when a new scan is taken) or is completed, a decision has to be made. Our proposed decision support algorithm tackles this challenging setting by suggesting the next action to be taken based on the global optimization objective(s).

At the core of our approach, we use a learning-based method to train a pairwise ranking function inspired by the learning-to-rank algorithm which is widely used in the information retrieval domain [22]. In our setting, namely assigning patients to medical professionals, we seek to learn the relative ranking over the available assignments. This ranking function is, in turn, used for comparing all possible patient–medical professional pairings and makes a selection based on a standard voting rule (e.g., majority vote). To that end, LBA first creates a large set of small-scale assignment scenarios based on relevant patients’ arrival models (which may be learned from past data, e.g., [34]) and available staff. These scenarios are (near) optimally solved using appropriate offline optimization techniques such as mathematical program solvers (e.g., Gurobi [35]), Monte-Carlo-Tree-Search (MCTS) methods [36] and others, given the institute’s objective. The optimized solution set is then translated into a set of training examples. Namely, each decision taken in the optimized solution is paired with all the other available decisions that were not taken, to create training instances. These instances are used to train the ranking function which is then used in the online setting by selecting the “best” decision after comparing all possible pairs of potential decisions (see Algorithm 1). Note, however, that our approach can be readily adjusted to provide a prioritized list of the available decisions (or, for that matter, the *top-k* decisions) rather than just the highest ranking decision. For simplicity and evaluation purposes, we focus on the latter case.
**Algorithm 1** The Learning-Based Assignment Process.1:Create a set of patient to medical professional assignment scenarios.2:Translate each scenario into an appropriate offline optimization problem.3:Solve (either optimally or sub-optimally) the offline optimization problems.4:Translate each optimized offline decision into a training instance.5:Train a ranking function.6:Use the resulting ranking function as an online policy.

Next, we apply and evaluate our approach in two medical domains: EDs and medical scan readings.

## 4. Implementation

We implement our approach while focusing on two medical settings: (1) the patient–physician matching in EDs; and (2) the exposure of scans to radiologists.

For each domain, we first discuss its distinctive characteristics pertaining to our approach. Then, we formalize the key factors associated with the domain. Next, we discuss the available data used in our implementation for generating offline scenarios. Then, we formulate the relevant offline optimization problems and discuss the appropriate solution procedure. Then, we present the training of our machine learning algorithm and its architecture, which, in turn, translates into an online policy. Last, we evaluate the learned policy using real-world data and discuss the results.

### 4.1. Patient–Physician Matching in EDs

Nearly half of all US hospital-associated medical care is delivered by EDs, also known as emergency rooms, making EDs a major source of medical care, especially for vulnerable populations [37,38]. EDs are faced with a dynamic flow of patients who present a wide variety of conditions, ranging from severe multiple percussive injuries and drug overdoses to common colds and cuts and scrapes, all of which seek fast and quality medical attention. Due to the variability in patients’ conditions, as well as the limited availability of medical resources and their own variability (i.e., attending physicians, interns, etc.), an efficient patient–physician matching is needed, a process which is often referred to as triage [39].

**Figure 1:**
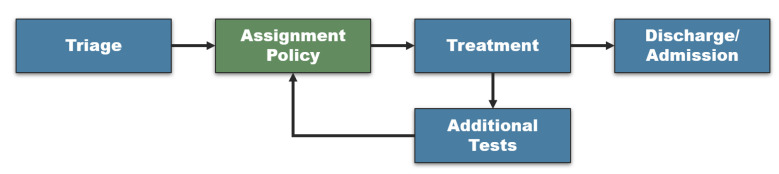
The common ED work-flow.

While different EDs deploy slightly different modes of operation, a basic work-flow is common to most modern EDs [40,41], as depicted in Figure 1. In words, when a patient arrives at the ED, her first stop would be the triage station, where she would receive a severity rank. Based on a assignment policy, which is in the focus of this study, the patient would then continue to a (first) treatment/examination by a medical professional. If the condition is appropriately diagnosed and/or treated, the patient may be released or admitted to a hospital ward. Otherwise, additional lab tests (e.g., CT, bloodwork) would be needed which in turn would require a re-evaluation of the patient’s severity rank and a second treatment/examination, possibly by a different physician. According to ED professionals, it is extremely rare for a patient to have more than two cycles of treatment before she is discharged or admitted. Our trained algorithm is intended to assist the triage nurse by advising on the best projected assignment.

The patient–physician matching is directed at getting the right patient to the right physician at the right time, given the ED’s constraints. Specifically, given a preliminary evaluation of the patient upon arrival (commonly done by a triage nurse) and the available medical staff, a decision has to be made as to *when* the patient should receive treatment and by *which physician*. Currently, the patient–physician assignment process focuses almost entirely on assigning each patient a severity level using triage scales (e.g., between 1 and 5, 1 being the highest [42]), which in turn translates into an *upper bound* on the desired patient’s waiting time, leaving the decision as to *when and which* medical professional should provide the treatment entirely in the hands of the triage nurse(s). Unfortunately, due to the time-critical environment, the multiple partially-conflicting objectives of the ED (as discussed next) and multiple interruptions, decisions are often inadequately made and are mainly based on conventional scheduling heuristics and experience which do not necessarily fully align with optimizing the ED’s objectives (e.g., [4,5,6]). Specifically, while EDs have been computationally investigated for over 70 years [43], mainly focusing on modeling the patient arrival flow and required staffing levels, to the best of our knowledge, the patient–physician assignment problem has yet to be addressed by computational means. To address this shortcoming, we make use of our approach (Section 3) and adapt it to the ED domain.

Our approach provides the ED with an effective and efficient policy targeted at optimizing the hospital-specific objectives given the hospital’s available resources and expected patient flow.

To ensure the validity of the application of our approach to the ED domain from a medical perspective, we recruited four medical professionals (who did not co-author this article), namely a triage nurse, a physician’s assistant, an attending physician and an ED director, from three large hospitals in Israel. We refer to these medical professionals as the *expert panel* in this study.

#### 4.1.1. Creating Patient–Physician Assignment Scenarios

Drawing on extensive modeling research based on data collected in one of the largest hospitals in Israel, *Rambam* hospital in Haifa [34], we are able to generate scenarios matching the real world. Specifically, the patient arrival process and physician’s and lab tests’ required time, among others, are modeled.

Rambam hospital works in a three-shift workday. For this evaluation, we focus on perhaps the most challenging shift—the *night shift*. The night shift takes place from 23:00 to 7:00 during which only 2–4 physician, of various seniority and specialties, work the ED, making the assignment extremely complex.

#### 4.1.2. The Optimization Problem

We start by modeling the two main sets of actors in the ED, patients and physicians and their interaction. Our modeling is based on existing literature and common clinical practices as prescribed by the expert panel.

Patients. A patient $p_{i}\in P$ is represented as a pair 〈Severity,Injury〉 where Severity is defined using a common triage scale (such as the popular Emergency Severity Index (ESI) [5]) (Using the ESI, each patient is assigned a number between 1 and 5 representing the acuity of her condition (with 1 being the most acute).) and Injury, which defines the type of injury or condition based on the patients’ symptoms (e.g., *Orthopedic*, *Internal*, etc.). According to clinical guidelines, $p_{i}$ is associated with the maximal time she is permitted to wait for the initial physician’s treatment, denoted $\bar{t_{p_{i}}^{1}}$ and, if needed, the second physician’s treatment $\bar{t_{p_{i}}^{2}}$. Patients may arrive at time *t* to the ED based on an estimated distribution $D_{p_{i}}\left(t\right)$, commonly assumed to follow an estimated distribution learned from past data [34,44]. We assume that, upon arrival to the ED, $p_{i}$’s characteristics are correctly identified by the triage nurse. After leaving the triage station (or when $p_{i}$’s test results arrive, see Figure 1), she is scheduled to meet one of the physicians $c_{j}$.

Physicians. A physician $c_{j}\in C$ is represented as a pair 〈Seniority,Specialty〉: Seniority is defined based on the physician’s qualifications over a discrete set (In Israel, as in most countries), *c*’s Seniority is classified to one the following (from lowest to highest): physician’s assistant, intern, resident and attending physician.) and Specialty indicates if the physician has “special training” in a specific injury type defined over the same set of injury types which characterize the patients (*NONE* otherwise). As a result, different physicians may have different required treatment times and varying levels of care quality. The set of available physicians, as well as their characteristics, is assumed to be known in advance and does not change during a shift.

Objective. The principal purpose of the ED is to ensure that patients receive the level and quality of care appropriate to their clinical needs and that the ED resources are most usefully applied to this end [45]. Unfortunately, explicitly quantifying the above purpose is highly complex [46], which often leads hospitals and governmental agencies to define multiple, often partially conflicting, objectives [47]. These objectives primarily focus on minimizing the following measures: (1) risk of adverse consequences to patients [48] (e.g., misdiagnosis, inappropriate medication); (2) ED over-crowdedness [49]; (3) interruptions to physicians [50]; (4) patients’ wait times [51]; and (5) patients’ length of stay in the ED [52].

With the help of the expert panel (who are familiar with Rambam hospital’s practices and Israel’s guidelines), we instantiate the ED’s objective and constants of our model which are not directly observable from data. Namely, we formulate the primary ED objectives as follows:

(1) Minimizing risk of adverse consequences. Each examination (e∈{1, 2}) performed by $c_{j}$ on $p_{i}$ has some risk of an adverse consequence. For example, a physician’s assistant with no specific specialty may be very well equipped to perform a first examination of minor orthopedic injuries with only a minimal risk of an adverse consequence, while severe head injuries should be examined by a qualified physician. We use $AC(p_{i},c_{j},e)$ as an indicator of whether $p_{i}$ was examined by $c_{j}$ in her *e*th examination. $risk_{i,j,e}$ denotes the risk of adverse consequences associated with such an examination.

(2) Minimizing patients’ waiting time. Each patient $p_{i}$, has to wait for her first (and second) examination for $WT(p_{i},e)$ minutes. Given $p_{i}$’s $\bar{t_{p_{i}}^{1}}$ and $\bar{t_{p_{i}}^{2}}$ (as defined by the triage scale), the ED seeks to minimize the wait time and avoid exceeding the wait time limits. The penalty for exceeding the limits is provided by $exc_{i,e,\delta_{t}}$ where $\delta_{t}$ is the excess wait time.

(3) Minimizing patients’ length of stay. Each patient $p_{i}$ spends $LOS(p_{i})$ minutes from the time she arrives at the ED to the time she is discharged or admitted to a hospital ward. This $LOS(p_{i})$ includes the time $p_{i}$ waits for examinations, the treatment time needed by the physicians, denoted $CE\left(c_{j}\right)\cdot TT\left(p_{i},e\right)$ and (if needed) lab test time $LT(p_{i})$ between the two examinations. $TT(p_{i},e)$ denotes the nominal treatment time and $CE(c_{j})\geq 1$ denotes the physician’s time efficiency factor, capturing the relative “examination speed” which varies between physicians.

(4) Minimizing over-crowdedness. At any point in time, one can measure the number of patients currently waiting and being treated in the ED, denoted κ(t), where *t* indicates continuous time.

(5) Minimizing interruption to physicians. Unfortunately, in some (extreme) cases, a physician may be asked to stop the treatment of one patient in order to treat another. This *preemption* may be very costly. The number of preemptions during $p_{i}$’s *e*th examination is denoted as $PC(p_{i},e)$. The penalty for each interruption is given by $pre_{i,e}$.

The ED must choose, for each patient $p_{i}$, which physician $c_{j}$ will provide the examination/treatment *e* and at what time *t*. Let $y_{j}^{i}\left[e,t\right]$ be indicator decision variables denoting that patient $p_{i}$ is assigned to physicians $c_{j}$ for her *e*th examination at time *t*. We assume that the ED is evaluated based on some metric, defined by stakeholders and governmental agencies, over the above five objectives, e.g., using a linear objective which summarizes the weighted objectives over all patients, examinations and time as proposed in Equation (1). Note, however, that the objective need not be linear, and, in the interest of generality, we do not assume it to be in the following sections.

(1) $$\begin{array}{l} \underset{y_{j}^{i}\left(e,t\right)}{Minimize}\sum_{p_{i}}\sum_{e\in \{1,2\}}\sum_{c_{j}}(\int_{t}y_{j}^{i}\left[e,t\right]\\ \;(\alpha_{1}AC\left(p_{i},c_{j},e\right)risk_{i,j,e}+\alpha_{5}PC\left(p_{i},e\right)pre_{i,e})dt+\\ \;\alpha_{2}WT\left(p_{i},e\right)exc_{i,e,\delta_{t}}+\alpha_{3}LOS\left(p_{i}\right))+\alpha_{4}\int_{t}\kappa \left(t\right)dt) \end{array}$$

Table 1 summarizes the paper’s notations.

When the patients’ arrival times and characteristics are known in advance, optimal patient–physician assignment can be derived over a discrete finite horizon t=0,…,T using the following Mathematical Problem (MP):

(2) $$\begin{array}{c} \begin{array}{c} \underset{y_{j}^{i}\left[e,t\right]}{Minimize}\;\mathrm{ED}\;\mathrm{Objective}\;(i.e.,\;\mathrm{Equation}\;(1)) \end{array} \end{array}$$

(3) $$\begin{array}{cc} s.t & \sum_{i}\sum_{e}y_{j}^{i}\left[e,t\right]\leq 1\;\forall j,t \end{array}$$

(4) $$\begin{array}{cc} \; & \sum_{j}y_{j}^{i}\left[e,t\right]\leq 1\;\forall i,e,t \end{array}$$

(5) $$\begin{array}{cc} \; & \rho_{i,j,e,t}=y_{j}^{i}\left[e,t\right]\left(\neg y_{j}^{i}\left[e,t-1\right]\right)\;\forall i,j,e,t\geq 1 \end{array}$$

(6) $$\begin{array}{cc} \; & \chi_{i,e,t}=t\cdot \sum_{j}\rho_{i,j,e,t}\;\forall i,e,t\geq 1 \end{array}$$

(7) $$\begin{array}{cc} \; & st_{i,e}=\min_{t}\left\{\chi_{i,e,t}\right\}\;\forall i,e \end{array}$$

(8) $$\begin{array}{cc} \; & \phi_{i,e,t}=t\sum_{j}\left(\neg y_{j}^{i}\left[e,t\right]\right)y_{j}^{i}\left[e,t-1\right]\;\forall i,e,t\geq 1 \end{array}$$

(9) $$\begin{array}{cc} \; & et_{i,e}=\max_{t}\left\{\phi_{i,e,t}\right\}\;\forall i,e \end{array}$$

(10) $$\begin{array}{cc} \; & st_{i,e}<et_{i,e}\;,\;st_{i,2}\geq et_{i,1}\;\forall i,e \end{array}$$

(11) $$\begin{array}{cc} \; & {\mathrm{arrival}}_{i,2}=et_{i,1}+LT\left(p_{i}\right)\;\forall i \end{array}$$

(12) $$\begin{array}{cc} \; & st_{i,e}\geq {\mathrm{arrival}}_{i,e}\;\forall i,e \end{array}$$

(13) $$\begin{array}{cc} \; & LOS\left(p_{i}\right)=et_{i,2}-{\mathrm{arrival}}_{i,1}\;\forall i \end{array}$$

(14) $$\begin{array}{cc} \; & WT\left(p_{i},e\right)=st_{i,e}-{\mathrm{arrival}}_{i,e}\;\forall i,e \end{array}$$

(15) $$\begin{array}{cc} \; & \sum_{j}\sum_{t}y_{j}^{i}\left[e,t\right]/CE\left[j\right]=TT\left[p_{i},e\right]\;\forall i,e \end{array}$$

(16) $$\begin{array}{cc} \; & PC\left(p_{i},e\right)=\sum_{t}\sum_{i}\rho_{i,j,e,t}-1\;\forall i,e \end{array}$$

(17) $$\begin{array}{cc} \; & AC\left(i,j,e\right)=I\left(\sum_{t}\rho_{i,j,e,t}>0\right)\;\forall i,j,e \end{array}$$

The MP consists of an ED-specific objective function (e.g., Equation (1)) and the following constraints: Equations (3) and (4) enforce that at most one patient is treated at a time by each physician and, similarly, at most one physician can treat a patient at a given time. Equations (5)–(7) extract the treatment start time and Equations (8) and (9) extract the treatment end time. Equations (10) and (12) enforce a valid treatment duration, while Equation (11) extracts the time in which a patient becomes available for her second treatment. *Note that* ${\mathrm{arrival}}_{i,1}$ *is assumed to be given in the offline setting*. Equations (13) and (14) extract each patient’s LOS and WT, respectively. Next, Equation (15) makes sure that the time physicians are assigned to a patient is appropriate and Equation (16) extracts the preemptions that took place. For simplicity, the above MP assumes each patient is treated twice.

Note that the patient–physician assignment problem is akin to the well studied job shop scheduling problem of unrelated machines with preemption, with the analogy of physicians to machines and patients to incoming jobs, as discussed below. This problem is known to be NP-hard [53].

#### 4.1.3. Solving the Offline Optimization Problems

To solve the offline optimization instances, we use a Mixed Integer Linear Program (MILP) available at https://goo.gl/rXaBRh (accessed on 25 April 2021).

#### 4.1.4. Training Instances

Using the set of optimized solutions generated offline, we identify the times at which a new patient arrives or when treatment of a patient is completed. For each such case, we create all pairs consisting of the selected assignments according to the optimized solution ($\langle p_{i}^{☆},c_{j}^{☆}\rangle$ or $\langle p_{i}^{☆},WaitRoom\rangle$) coupled with any other assignment option which was not selected (i.e., $\langle p_{i}^{☆},c_{j}\rangle$ or $\langle p_{i},c_{j}^{☆}\rangle$ and WaitRoom options). For simplicity, from this point onwards, we consider the assignment to the WaitRoom as a dummy physician which can support an infinite number of patients but does not provide any treatment. The resulting pairs are used as training data for a supervised ranking machine learning algorithm as we discuss next. In other words, we use the set of optimized solutions to generalize and mimic the optimal decisions made in the offline settings.

With the help of the expert panel, we define a feature vector that combines a description of the patient and the physician’s current state, as shown in Table 2.

#### 4.1.5. Ranking Function

Inspired by the neural network ranking approach in information retrieval [22], we develop a new Deep Neural Network (DNN) architecture targeted at learning to rank among assignments based on the created dataset of pairs discussed above.

**Figure 2:**
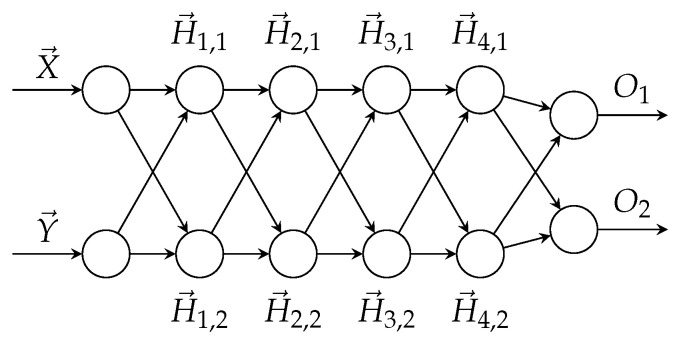
DNN comparator.

Specifically, our DNN is composed of two identical sub-networks, with shared weights. The network architecture is shown in Figure 2.

The anti-symmetric nature of the network is built by sharing weights, as can be demonstrated for the connection between the input and the first hidden layer:

$$\begin{array}{cc} {\vec{w}}_{i,1}^{\;1} & =w\left(\vec{X}\to \vec{H_{1,1}}\right)=w\left(\vec{Y}\to \vec{H_{1,2}}\right)\\ {\vec{w}}_{i,1}^{\;2} & =w\left(\vec{X}\to \vec{H_{1,2}}\right)=w\left(\vec{Y}\to \vec{H_{1,1}}\right) \end{array}$$

The bias term of both parts of the first hidden layer is also shared. Thus, the two output vectors of the first hidden layer are:

$$\begin{array}{cc} \vec{H_{1,1}} & =\tanh({\vec{w}}_{i,1}^{\;1}\cdot \vec{X}+{\vec{w}}_{i,1}^{\;2}\cdot \vec{Y}+\vec{b_{1}})\\ \vec{H_{1,2}} & =\tanh({\vec{w}}_{i,1}^{\;1}\cdot \vec{Y}+{\vec{w}}_{i,1}^{\;2}\cdot \vec{X}+\vec{b_{1}}) \end{array}$$

The rest of the layers share weights and connections in a similar fashion with their appropriate activation functions. Complete technical details are available in our code.

This architecture has the following properties:

1. *Reflexivity*: For identical input vectors, the network produces identical outputs.
2. *Anti-symmetry*: For input vectors x,y, if x≻y (reads “*x* is preferred over *y* according to the DNN”), then, for input vectors y,x, we get y≺x and vice versa.

These properties make the network well suited to learn pairwise ranking functions.

#### 4.1.6. Online Policy

The trained DNN is used in the online phase of the algorithm as follows: At each assignment event, i.e. patient arrival, or the completion of treatment, all possible assignments are compared to each other. Each comparison is worth one point to the higher ranking assignment. Using a majority vote with random tie-breaking, the patient–physician assignment is selected and presented to the user (i.e., the triage nurse).

#### 4.1.7. Experimental Results

To evaluate our approach, we use real-world data and compare our LBA approach with the conventional online heuristic deployed in EDs. This heuristic is often known as First-Come-First-Served-with-Urgencies (*FCFSwU*) which, according to our expert panel, is the backbone of most ED assignment decisions, including those at Rambam hospital. FCFSwU works as follows: patients of severity Levels 3–5 are treated as a single “non-urgent” type and are admitted in a first-come-first-served fashion to a physician who specializes in the relevant injury type or to a physician with no specialty. Specifically, a patient would not be assigned to a specialist who specialized in a different injury type. Patients of severity Level 1 or 2 are treated as a single “urgent” type and, upon arrival, the most senior specialized physician who is not already treating another urgent patient is called (or interrupted) in order to provide the needed treatment.

We examined two scenarios: *normal* patient flow and *heavy* patient flow. The normal patient flow is provided in the literature [34], whereas the heavy patient flow is derived by multiplying the distribution parameters, resulting in twice the number of patients on expectancy. We trained two LBA DNNs, one for the normal patient flow case, denoted $LBA_{N}$, and one for the heavy patient flow case, denoted $LBA_{H}$.

We randomly generated 500 scenarios for each flow type, which in turn were optimally solved using the Gurobi solver [35]. The results are translated into two training sets for $LBA_{N}$ and $LBA_{H}$. We evaluated both $LBA_{N}$ and $LBA_{H}$ versus the FCFSwU heuristic on a series of 100 simulations of eight-hour shifts sampled according to the parameters discussed earlier. Both approaches were evaluated using the expert panel objective function available in our code.

Interestingly, for all 100 sampled instances, the LBA approach outperforms the FCFSwU heuristic. The difference is statistically significant, for both the normal and heavy patient flow types, using a paired samples *t*-test, p<0.05.

We further evaluated the results based on the five major ED objectives. We encounter the following results:

1. Risk of Adverse Consequences: The average risk of adverse consequences was reduced by 10% (normal flow) and 15% (heavy flow) compared to FCFSwU.
2. Wait times: The average wait time was slightly reduced by an average of 20 s per patient, across both patient flow conditions, compared to FCFSwU.
3. Length of stay: The average length of stay was reduced by 5% (normal flow) and 11% (heavy flow) compared to FCFSwU.
4. Crowdedness: There were no significant differences.
5. Interruptions: The most prominent difference was measured in the number and cost of preemptions. Specifically, a treatment is approximately 10 times more likely to be interrupted using the FCFSwU compared to LBA. By weighting the interruptions by their associated penalties, we see a penalty that is 20 times higher per shift.

Table 3 summarizes the results.

The results further show that *there is no apparent trade-off between the LBA approach and the FCFSwU heuristic*. Specifically, based on the results, the use of LBA improved four out of the five performance metrics while having no impact on the fifth. Namely, our approach provides a Pareto improvement over the existing practices.

All code and data used for evaluating our approach in this domain are available at https://goo.gl/rXaBRh (accessed on 25 April 2021).

### 4.2. Radiology

#### 4.2.1. Medical Imaging

Medical imaging is a key diagnostic tool for many diseases and plays an important role in monitoring treatment and predicting outcomes [54]. Medical images, such as MRI, CT, ultra-sound, etc., are transferred digitally to a shared storage which is accessible for the radiologists (also known as *readers*). The readers, in turn, select the images (also known as “studies”) they would like to work on (also known as “reading”).

From a medical perspective, each study is associated with a soft-deadline for completing the reading of the study. Violation of the deadline increases the patient’s risk of medical complications and might prolong her suffering. These adverse consequences are greatly exacerbated as the tardiness is increased. Thus, medical institutions apply a study-to-reader exposure policy aimed at minimizing overly tardy study readings. This exposure policy controls, for each study, the subset of readers who will have access to it. This exposure may vary over time as new studies arrive while the tardiness of others increases. The rationale of this policy is to have the ability to expose urgent studies (or a given study which has become urgent) to a wider group of readers.

Naïve exposure polices, such as exposing all studies to every reader (maximum freedom for readers) or exposing a single study to each reader (no freedom), are not practical due to information overload on one hand and lack of autonomy on the other hand (which is not not acceptable by today’s medical imaging standards). Deriving an efficient study-to-reader exposure policy is extremely difficult due to two main factors:

1. The inherent uncertainty in the arrival times of new studies.
2. The local nature of the readers’ decisions may not align with the global objective.

To address these difficulties, we make use of our approach (Section 3) and adapt it to the medical imaging domain. Note, that unlike the ED domain, radiologists expect to have a greater degree of freedom in prioritizing their work.

To ensure the validity of the application of our approach to the medical imaging domain from a medical perspective, we recruited a domain expert (who do not co-author this study). The expert has been working with several large medical institutions in Israel and abroad.

#### 4.2.2. Creating Study-Reader Assignment Scenarios

For evaluating our approach, we used extensive data collected over a three year time span from a major medical institution in Israel (to remain anonymous). The medical institution employs more than 40 readers handling thousands of studies a year from several physical sites. The institution deploys a naïve exposure policy, exposing all studies to all readers, and has thus requested our help.

#### 4.2.3. The Optimization Problem

In our setting, there are two main sets of actors: studies and readers. Our model is based on common clinical practices as prescribed by a domain expert (who did not co-author this paper).

**Studies.** A study $s_{i}\in S$ is represented as a tuple 〈M,B,D〉 where *M* (Modality) is defined over a finite set consisting of the available imaging devices (e.g., MRI, CT, etc.) and *B* is taken from the set of body parts. *D* (Deadline) represents the maximal desired time in which the reading of the study should be completed. For each study $s_{i}=\langle M,B,D\rangle$, the nominal time for a reader to read $s_{i}$ is given by T(M,B). We assume that studies arrive according to a known distribution.

The stochastic arrival of studies is assumed to follow a non-homogeneous Poisson process, as established in the past for various medical arrival processes [34]. The parameters of the arrival model were estimated via maximum likelihood estimation and provided to us by the domain expert.

**Readers.** A reader $r_{j}\in R$ is represented by Subspeciality which is a list of all 〈M,B〉 pairs for which he is considered an expert. Note that every reader is qualified to read any study; however, reading a study outside his expertise takes longer. Formally, if reader $r_{j}$ does not possess the required sub-speciality for study $s_{i}$, the time needed for reading $s_{i}$ would be the nominal time given by *T* multiplied by a skill-efficiency factor ρ where ρ>1.

Since readers expect to select the studies they wish to work on from their exposed list, we model their preference as a utility function $u_{j}:S\to R^{+}$. Due to the time constraints and the high work load, it is reasonable to assume that readers follow a quantal response decision-making [55]; specifically, the probability that reader $r_{j}$ will select study $s_{i}$ from his pool ($S_{j}$) is defined as

$$p\left(r_{j},s_{i}\right)=\frac{e^{u_{j}\left(s_{i}\right)}}{\sum_{s_{k}\in S_{j}}e^{u_{j}\left(s_{k}\right)}}$$

Eliciting the actual utilities of readers is extremely complex, therefore we follow the revealed preference theory [56] and rely on historical data to model $p(r_{j},s_{i})$. From the data which were made available to us by the institute, we extracted the set of studies to which each reader was exposed and grouped them by 〈M,B〉. Then, the normalized frequency of selected 〈M,B〉 by each reader was used as a proxy for that reader’s utility. This utility proxy was then used within a quantal response model, as specified in Section 4.2.2.

**Objective.** As discussed above, each study $s_{i}$ is associated with a soft deadline (*D*). Medical institutions strive to minimize the tardiness of the studies. More specifically, they seek to avoid overly tardy studies. For example, according to medical experts, it is preferable to have two studies read one hour past their deadline than to have one study read two hours past its deadline.

Formally, given the above characteristics, we define a time-based loss function $L(\left\{{\mathrm{lateness}}_{i}\right\})\to R$ for all completed studies, where ${\mathrm{lateness}}_{i}$ is defined as the difference between the time $s_{i}$ was read and its deadline. Unlike tardiness, which can only take positive values, lateness can be either negative (indicating that the study was read before its deadline had passed) or positive (indicating that the study was read after its deadline). The loss function L should obey the following criteria:

1. **Additivity**: $L\left(\left\{{\mathrm{lateness}}_{i}\right\}\right)=\sum_{i}L\left({\mathrm{lateness}}_{i}\right)$, giving equal weight to the loss contribution of all studies.
2. **Super-Linearity**: L∈ω(lateness), i.e., ∀α≥1.L(αl)≥αL(l) capturing the preference described above, namely, L(1)+L(1)≤L(2).
3. **Non-negativity**: ∀l.L(l)≥0 insuring that an early reading of a study does not “compensate” for the loss incurred by the tardy reading of another study.

These criteria define a family of functions capturing the varying preferences of the medical institutions.

Obeying the definitions above, and with the help of our domain expert, we define the following loss function:

$$L\left(s_{i}\right)=\exp\left(2\cdot \mathrm{lateness}{}_{i}\right)$$

Since L is additive, the total loss is

$$L\left(S\right)=\sum_{s_{i}\in S}L\left(s_{i}\right)$$

where lateness ${}_{i}$ is measured in hours.

**Figure 3:**
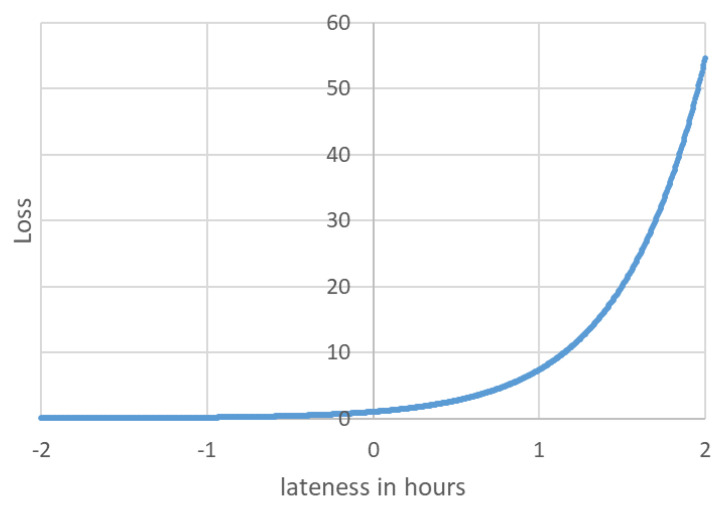
Loss as a function of lateness.

The loss function is shown in Figure 3 for the range of [−2,2] h around the deadline. As shown in the figure, the loss encountered by studies read one hour or more after their deadline is significantly higher then those read earlier.

Note that our approach provides stakeholders with the flexibility of specifying any loss function of their choice, potentially even those which do not adhere to the three criteria defined above.

The institute must choose, for each scan $s_{i}$, which readers $r_{j}$ will be exposed to $s_{i}$ at time *t*. Recall that in the ED domain, once a decision has been made, the outcome is deterministic. In contrast, here, the (partial) autonomy of the readers introduces stochasticity into the assignment process. Namely, for each decision, there are several possible outcomes depending on the readers’ decisions since it is likely that most studies are exposed to more than a single reader. This, in turn, makes standard solvers practically inapplicable. In other words, even if the arrival time and characteristics of each $s_{i}$ is known in advance, an optimal offline policy is hard to derive. We tackle this challenge by utilizing the Monte Carlo Tree Search (MCTS) [36] method and adapt it to our setting as discussed next.

Table 4 summarizes the key notations.

#### 4.2.4. Training the Ranking Function

To address the stochasticity in our setting, we propose to merge Steps 3–5 of our approach (Section 3), by utilizing the popular optimal policy approximation MCTS algorithm. MCTS uses exploratory traversal of the search space using many simulations to approximate an optimal policy [36]. MCTS has proven itself capable of achieving state-of-the-art performance in modeling both online dynamic behavior and the long-term effect of an agent’s decisions in complex environments such as the games of Go and Chess [57]. To that end, we first sample from a study arrival model a set of scenarios consisting of the study arrivals, a set of readers and their associated probabilistic preferences.

Then, an MCTS algorithm is used to approximate the optimal ranking policy aimed at minimizing L. Formally, when a new study $s_{i}$ arrives, for each reader $r_{j}$, we compare $s_{i}$ to his lowest ranking pending study, denoted $s_{j}^{-}$. If $C(r_{j},s_{i},r_{j},s_{j}^{-})\geq 0.5$, then $s_{i}$ will replace $s_{j}^{-}$ and will be inserted in its correct order by using C as a partial-order operator on the reader’s studies using the reader’s preference. Similarly, when the reading of a study $s_{i}$ is completed by reader $r_{j}$, all studies which are not exposed to $r_{j}$ are ranked using C, and the highest ranking study is added to $r_{j}$’s list. This procedure is executed in parallel for all available readers.

We use a deep-learning-based architecture to represent C. To enable learning and generalization, each 〈study, reader〉 pair is represented by a unified feature vector as outlined by the domain expert. The features, which we consider in modeling a reader at a specific moment in time, are: his subspecialty, the percentage of studies which lay within his subspecialty in his pool and the number of studies which passed their deadline in his pool. The features we used in modeling studies are: the study’s lateness, the associated subspecialty of the study, time left for reading the study and to how many readers the study is exposed. Overall, each 〈study, reader〉 pair is represented by seven features.

We use the same architecture discussed in Section 4.1.5, which we demonstrated to guarantee anti-symmetry (i.e., if $s_{i}$ is strictly preferred to $s_{k}$ then $s_{k}$ is not strictly preferred to $s_{i}$) and reflexivity (i.e., $s_{i}$ is weakly preferred to $s_{i}$). The network architecture is depicted in Figure 2.

#### 4.2.5. Online Policy

The trained comparator C is used to update the readers’ exposure list at the arrival of a new study and at the completion of reading.

Algorithm 2 summarizes the adaption of our approach (Section 3) to the medical imaging setting.
**Algorithm 2** Learning-Based Assignment with Exposure.1:Create a set of studies to readers scenarios.2:Apply MCTS in order to derive a policy from many simulations.3:Translate each decision made by the MCTS into training instances.4:Train a DNN comparator.5:Use the resulting DNN as an online exposure policy.

As the number of simulations performed on Line 2 of Algorithm 2 is increased, the resulting training samples cover a larger portion of the possible state-space, gradually approximating the optimal exposure policy.

#### 4.2.6. Experimental Results

The conventional exposure policy used by many medical institutions is to expose each study to all readers and let the readers select the studies they wish to read. We refer to this policy as the *Naïve* policy. As mentioned above, the *Naïve* policy suffers from two major limitations: The first and most obvious is the readers’ information overload. Second, and perhaps more surprising, is that the resulting lateness distribution of the studies is shown to have a long tail, meaning that some studies are disproportionately late.

The use of exposure policies has recently been proposed in medical institutions in order to mitigate the above limitations. The rationale behind these exposure policies is to expose urgent studies (or a given study which has become urgent) to a wider group of readers. A commonly applied heuristic, denoted *H*, divides the time interval between the arrival of a study and its deadline into three equal intervals: For the duration of the first interval, the study is exposed to readers with the appropriate subspecialty. For the second interval, the study is a wider group of readers (but not all of them). Finally, the study is exposed to all readers until it is read.

Note that neither policy limits the number of exposed studies per reader (*k*) and thus information overload may still pose a concern.

For training our DNN, we executed Algorithm 2 with the following parameters, consistent with our real-world data:

- *R* consists of 10 readers. The reader’s sub-speciality list is sampled according to the collected data. The reader’s processing time varies between 3 (if the study is within the reader’s sub-speciality) and 9 (otherwise). The preference model for the reader is set according to Section 4.2.3.
- *S* consists of 60 studies whose type, deadline and arrival time were sampled from the study arrival model. A study’s deadline is in the range of 30 min to 1 week.
- *k* was set to 10, based on our discussion with a domain expert.

The exposure policy prescribed by the trained DNN, C, was evaluated next.

We evaluated our exposure policy in extensive simulations comparing our policy with the two baseline policies, *Naïve* and *H*.

**Figure 4:**
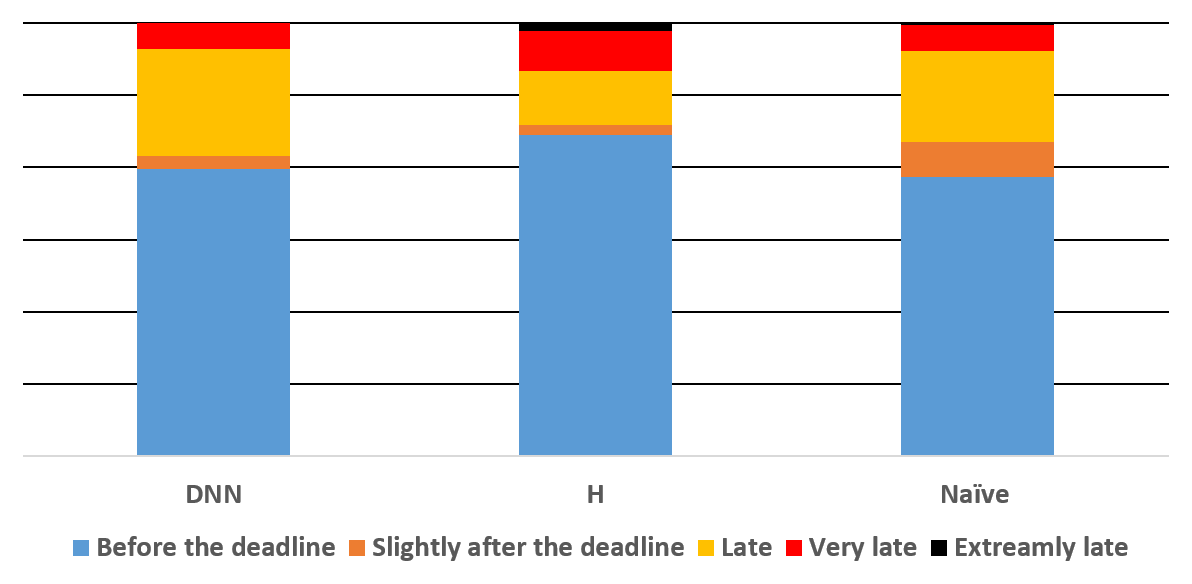
Lateness distribution for the three algorithmic solutions. Error bars are too small to be presented. Slightly over the deadline = less than 15 min after the deadline; Late = between 15 and 45 min past the deadline; Very late = between 45 and 60 min past the deadline; Extremely late = more than 1 h past the deadline.

Using the same parameters that were used for training, our learning-based exposure policy significantly outperforms the baseline policies in terms of the overall objective. Specifically, our approach achieved an average score of 69 compared to 82 and 75.5 achieved by *H* and *Naïve*, respectively (recall, the lower the better). The difference was found to be statistically significant in the p<0.05 range using a one-way ANOVA test followed by pair-wise comparisons using paired-sampled *t*-tests with the Bonferroni correction. Figure 4 and Table 5 present the lateness distribution under the three policies. The DNN algorithm is the only one with no study read “extremely late” (i.e., more than 60 min past its deadline).

Note that *H* provides a significantly larger portion of studies read before the deadline (74%) compared to the other approaches, p<0.05, using the same statistical analysis as before. However, at the same time, it further provides a significantly larger portion of studies read very or extremely late, p<0.05.

We further evaluated the resulting reader’s pool size, *k*, during the experiments. Following our proposed approach, *k* is constrained so as not to exceed 10, and in our experiments it averages close to 10 studies exposed to each reader with minimal variability. For the baseline models, the situation is different: the *Naïve* policy exposes an average of 25.5 studies per reader (s.d = 16) and *H* exposes 18 (s.d = 12). Namely, significantly larger pool sizes were recorded for the baseline models which, in turn, are also characterized by large variability. Note that high variability in the number of exposed studies may confuse the readers.

To assess the ability of our approach to generalize and perform well under diverse circumstances, we repeat the above experiment while varying the number of readers (|R|) and their study pool size (*k*). We set the number of readers to either 5,10 or 15 and k=2,5 or 10. Again, each setting was tested for 100 simulations. The results portray a similar image to the one presented before—our learning-based exposure policy significantly outperforms the baseline policies in terms of the overall objective. Specifically, our approach achieved an average score of 68 compared to 98 and 78 achieved by *H* and *Naïve*, respectively (recall, the lower the better). The difference is found to be statistically significant in the p<0.05 range using a one-way ANOVA test followed by pair-wise comparisons using paired-sampled *t*-test with the Bonferroni correction. Table 6 presents the lateness distribution under the three policies.

Note that *H* results in significantly more studies that are read prior to their deadline compared to the *Naïve* and DNN conditions. Nevertheless, this comes at a price—significantly more studies are read very late, bringing about a poor overall score.

When comparing our approach’s performance under varying *k* values, we see that the performance is improved for lower *k* values. Specifically, for k=15, it achieves an average score of 79.5, while, for k=2, it achieves an average of 59 (k=5:65, k=10:69). This result is not surprising since a lower *k* means less freedom for the readers and a higher level of control for the system. Specifically, setting k=1 means that we completely eliminate the readers’ autonomy. The fact that lateness is minimized when *k* is reduced is evidence of the generalization ability of our approach to capture the system-wide objective while only being trained with k=10.

## 5. Discussion

The results from both domains indicate that the use of the LBA approach encompasses significant benefits compared to the conventional medical assignment policies. Specifically, by leveraging real-world data and an explicitly defined complex objective, the LBA approach can bring about a better suited policy to both domains.

By explicitly considering the domain-specific objectives and constraints (e.g., arrival flow and available staff), LBA further allows stakeholders to experiment and investigate different operation modes, work load, staff shortages, etc., which are at the core of medical operations research and practice [43]. These may also include “softer” objectives (not usually specified by medical metrics) such as the fairness of a medical professional’s workload. Such an investigation could be accomplished by simply changing the modular functions and constants in the above formulations and re-running the LBA process.

The ability to control and maintain the exposure list size, as shown in the evaluation of our second setting, also enables our approach to strike a delicate balance between the readers’ desire for autonomy in the selection of studies and the overall objective. Maintaining small exposure list sizes is of great importance, as it is well known that large exposure lists result in an increased user decision-making time [58] and lower user satisfaction [59].

However, when presenting a new approach, such as LBA, it is worth discussing its limitations. First, the results demonstrate an interesting trade-off between performance and development time. While LBA allows for better performance, arriving at the LBA policy requires the construction of the instances, solving them and training a supervised learning model, which in turn takes significantly more time compared to the easy-to-deploy heuristic commonly applied today. It is important to note that the LBA training is performed *offline*; thus, in deployment of the resulting policy, no run-time differences are encountered. Second, deploying an automated policy, such as the one proposed in this study, may encounter deployment challenges or even resistance from the medical staff. We are currently working with the largest hospital in Israel to deploy our approach in their ED. We are also looking into the possibility of collaborating with an industrial partner in order to evaluate the medical imaging exposure policy in a hospital. Third, to deploy our approach, access to real-world data or statistical models are needed. If such data are not available, transfer learning [60] of datasets from similar domains may be applied. Fortunately, the abundance of data and their collection by medical institutions, which is a common practice today, mitigate this concern. Finally, our approach relies on explicit features for representing the decision points used by the supervised machine learning model. Since the two settings explored in this study could be easily represented using a limited number of features, the neural network was able to successfully capture their relevant dependencies. In more complex settings where the number of raw features is particularly large, automatic feature extraction methods (e.g., [61]) could be used and will be explored in future work.

## 6. Conclusions

This article introduces and extensively evaluates a novel decision support algorithm for assigning patients to medical professionals termed Learning-Based Assignment (LBA). LBA combines both machine learning and optimization techniques in order to learn an efficient online policy. We demonstrate the benefits of the LBA approach in two medical domains and show that it outperforms existing practices.

We plan to extend this work in three main directions: First, since many hospitals also operate as training centers, there may also be an added value for assigning multiple physicians of different seniority to treat the same patient in an ED setting. Therefore, we plan to extend our model to incorporate these complex allocation objectives. Second, we plan to apply our approach to additional domains such as recommending tasks to workers in crowd-sourcing platforms (e.g., Amazon Mechanical Turk (https://www.mturk.com/, accessed on 25 April 2021). Effective, online policies play a critical role in such web-based systems and may benefit both the system’s owner by maximizing productivity and the workers themselves by enhancing their experience with a small set of highly matching task options. In the analogy to this article’s setting, tasks (in our setting, studies) arrive stochastically to the crowd-sourcing platform (shared pool); workers (readers) display different preferences over the tasks; and there is some benefit/loss associated with different workers performing different tasks at different times.

In addition, as mentioned above, we are currently working with the largest hospital in Israel to deploy our approach in their ED. We are also looking into the possibility of collaborating with an industrial partner in order to evaluate the medical imaging exposure policy in a hospital.

We hope that this study will encourage other researchers to tackle the important and challenging task of promoting quality and timely medical care.

## Figures and Tables

**Table 1:** Summary of key notations.

Notation	Meaning
*t*	Time.
$p_{i}\in P$	Patient.
$c_{j}\in C$	Physician.
$AC(p_{i},c_{j},e)$	Indicator whether $c_{j}$ is assigned to
	$p_{i}$’s *e*th examination.
$WT(p_{i},e)$	$p_{i}$’s waiting time for her *e*th examination.
$TT(p_{i},e)$	Time (nominal) required for the
	*e*th examination of patient $p_{i}$.
$LT(p_{i})$	Time required for lab tests of $p_{i}$.
$CE(c_{j})$	A type-*c* physician time efficiency factor.
κ(t)	#patients in the ED at time *t*.
$PC(p_{i},e)$	#interruptions to $p_{i}$’s *e*th examination.

**Table 2:** Combined patient–physician feature vector.

Feature Vector		
Patient	severity (Following ESI)	1/5,2/5,…,5/5
	injury	one-hot vector
	remaining treatment time	in minutes
	wait time	in minutes
	remaining time	in minutes
Physician	seniority	1/4,2/4,…,4/4
	specialization	one-hot vector
	status	0-idle; severity of patient
	idle time	in minutes

**Table 3:** Marginal improvement of the LBA approach compared to FCFSwU.

Criteria	Normal Load	Heavy Load
Risk	10%	15%
Wait times	marginal	marginal
Length of stay	5%	11%
Crowdedness	-	-
Interruptions	95%	90%

**Table 4:** Summary of key notations.

Notation	Meaning
$s_{i}\in S$	Studies
$r_{j}\in R$	Readers
${\mathrm{latness}}_{i}$	Current time—$s_{i}$’s deadline
*k*	Number of exposed studies per reader
ρ	Skill-efficiency factor
$p(r_{j},s_{i})$	Probability that $r_{j}$ will select $s_{i}$ from his pool
L	Loss function

**Table 5:** Lateness distribution for the three policies for 10 readers and k=10.

Algorithm	*DNN*	*H*	*Naïve*
Before the deadline	66.5%	74%	64%
Slightly after the deadline	3%	2.5%	8%
Late	25%	12.5%	21%
Very late	5.5%	9.5%	6.5%
Extremely late	0%	1.5%	0.5%

**Table 6:** Lateness distribution for the three policies.

Algorithm	*DNN*	*H*	*Naïve*
Before the deadline	66.5%	74.5%	72%
Slightly after the deadline	3%	4%	4%
Late	25%	18%	21%
Very late	5.5%	0.5%	1.5%
Extremely late	0%	3%	1.5%

## Data Availability

Not applicable.
